# Morphological Evidence for a Unique Neuromuscular Functional Unit of the Human Vocalis Muscle

**DOI:** 10.3390/ijms252211916

**Published:** 2024-11-06

**Authors:** Rareș-Vasile Tracicaru, Lars Bräuer, Michael Döllinger, Martin Schicht, Bernhard Tillmann, Delia Hînganu, Liliana Hristian, Marius Valeriu Hînganu, Friedrich Paulsen

**Affiliations:** 1 Department of Morphofunctional Sciences, Anatomy and Embryology, Grigore T Popa University of Medicine and Pharmacy Iași, University Street No 16, 700115 Iași, Romania; hinganu.delia@umfiasi.ro (D.H.); marius.hinganu@umfiasi.ro (M.V.H.); 2 Institute of Functional and Clinical Anatomy, Friedrich-Alexander-Universität Erlangen-Nürnberg, Universitätstraße 19, 91054 Erlangen, Germany; lars.braeuer@fau.de (L.B.); martin.schicht@fau.de (M.S.); friedrich.paulsen@fau.de (F.P.); 3 Laboratory for Computational Medicine, Division of Phoniatrics and Pediatric Audiology, Department of Otorhinolaryngology Head & Neck Surgery, University Hospital Erlangen, Friedrich-Alexander-Universität Erlangen-Nürnberg, Waldstr. 21, 91054 Erlangen, Germany; michael.doellinger@uk-erlangen.de; 4 Anatomical Institute, Christian-Albrechts-Universität of Kiel, Otto-Hahn-Platz 8, 24118 Kiel, Germany; bntill@t-online.de; 5 Department of Engineering and Design of Textile Products, “Gheorghe Asachi” Technical University of Iași, 700050 Iași, Romania; liliana.hristian@academic.tuiasi.ro

**Keywords:** larynx, laryngeal muscles, laryngeal nerves, electron microscopy, immunofluorescence, neural network, synapses, neuromuscular junctions, voice formation, neurolaryngology

## Abstract

Human vocalization is a complex process that is still only partially understood. Previous studies have suggested the possibility of a localized neuromuscular network of the larynx. Here we investigate this structure in human dissection specimens using multiple immunofluorescence and transmission electron microscopy (TEM). In the area of the pars interna of the thyroarytenoid muscle, muscle fibers are present that are clearly differentiated from skeletal or cardiac muscle cells and show an intermediate ultrastructure. In addition, intramuscular neurons are present that are detectable by both electron and fluorescence microscopy and may have a sensory function in a local neuronal network. Also, several types of sensory and motor synapses are detectable and distributed throughout the pars interna of the thyroarytenoid muscle, with multisynaptic muscle fibers being a common feature. These findings suggest the existence of a previously unrecognized type of muscle fiber coupled to an intramuscular neuronal network, the presence of which could explain functional peculiarities at the laryngeal level.

## 1. Introduction

**Figure 1:**
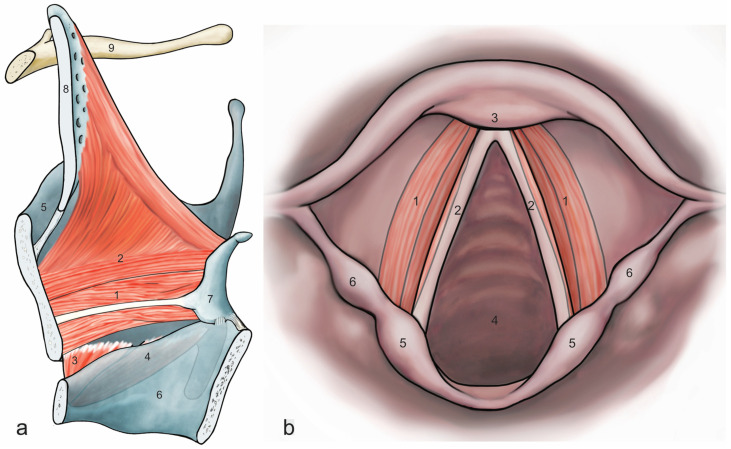
An overview of the main phonatory muscles. Image (**a**) is a sagittal view of the larynx showing the general disposition of the main phonatory muscles, the thyroarytenoid (TA), and the cricothyroid (CT): 1—internal part of the thyroarytenoid muscle (musculus vocalis), 2—lateral part of the thyroarytenoid muscle (pars muscularis), 3—vertical part of the cricothyroid muscle (pars recta), 4—(through transparency) oblique part of the cricothyroid muscle (pars obliqua), 5—thyroid cartilage, 6—cricoid cartilage, 7—arytenoid cartilage, 8—epiglottis, 9—hyoid bone. Image (**b**) is an axial view of the larynx at the level of the glottis. The internal part of the thyroarytenoid muscle (musculus vocalis) is seen lateral to the vocal cord: 1—internal part of the thyroarytenoid muscle (musculus vocalis), 2—vocal ligament, 3—thyroid cartilage, 4—glottis, 5—corniculate tuberculum, 6—cuneiform tuberculum. Artwork by illustrator Jörg Pekarsky of the Institute of Functional and Clinical Anatomy in Erlangen, Germany.

Vocal fold oscillation is an intricate process relying on complex activation patterns of the intrinsic laryngeal muscles [1,2]. Of these muscles, the cricothyroid (CT) and thyroarytenoid (TA) muscles (Figure 1a) have the greatest influence on the fundamental frequency [3] and the strongest correlation of the effects of their activity [1]. These muscles are known to be innervated from different sources, with the superior laryngeal nerve (SLN) innervating the CT and the recurrent laryngeal nerve (RLN) innervating the TA [4]. However, the results of nerve stimulation in the case of the RLN, which were obtained from the larynges of dogs, do not correspond linearly with the directly observed activation [5]. The same can be deduced from the non-linear relationship between the fundamental frequency (F_0_) and SLN stimulation [4]. This indicates that neural activity in these two muscles is dependent on more factors than previously assumed.

The laryngeal musculature has traditionally been considered skeletal muscle, although significant histologic [6] and molecular [7] differences have been pointed out in the past. The muscle fibers within the pars interna of the thyroarytenoid muscle (vocalis muscle) do not run parallel to the vocal ligament (VL). Instead, they run diagonally from the points of origin (the thyroid cartilage and arytenoid cartilage) in the direction of the VL. This creates a latticework of muscle bundles directly under the VL [6,8,9]. This finding also extends to the individual muscle fibers, where cross-linked fibers can be observed [6,10]. Even the presence of Purkinje-like cells has been described [6]. At the molecular level, recent studies have investigated the composition of myosin heavy chains (MHCs) in the larynx. In a previous study [11], we analyzed these results, including fibers containing all types of MHC (MHC 1, 2A, 2X). In particular, laryngeal muscle fibers often contain combinations of these myosin heavy chains (MHC 2-A-X), indicating multiple sources of innervation [11]. In fact, two or even three MHC types are usually co-expressed, suggesting that multiple innervation is a common occurrence [7]. The many studies of electrophoresis for these MHCs have led to the observation that a larynx-specific MHC is present in laryngeal muscles (MHC-L), which migrates between the bands for MHC-1 and MHC-2A on SDS-PAGE [12,13,14]. MHC-L is always expressed together with MHC-2A in fibers with a small cross-section and a high contraction velocity that show multiple innervation [12]. It is rapidly degraded in the absence of innervation (after excision) and is similar, but not identical, to the extraocular MHC (MHC-EO). This suggests that some muscle fibers in the larynx (about 8% according to D’Antona et al. [12]) have a particular type of innervation that determines the expression of this MHC.

In the innervation of the larynx, multiple endplates per muscle fiber are common [9,15], without a uniform distribution as observed in skeletal muscles [16,17]. Some authors have described spiral endplates together with the more classical en plaque endplates [18], mostly supplying thin extrafusal fibers. A connection with the MHC-L could be discussed, which was later found in similarly thin fibers. Muscle spindles are sparse and concentrated near the vocal folds [9,16], which express only limited nuclear pouch fibers, as no pouch-specific MHC was found [19]. However, the most interesting finding is the presence of intramuscular ganglia with pseudo-unipolar and bipolar neurons in all intrinsic muscles except the pars interna of the thyroarytenoid muscle (vocalis muscle) [16,20]. These express NOS but are not located near blood vessels, suggesting a sensory role [16].

Based on these findings, the neuromuscular innervation of the internal part of the CT muscle (vocalis muscle) and thus one of the two muscles central to phonation is still unresolved. To shed light on the distribution and properties of both the neural and muscular systems in the key region of the internal part of the TA muscle, we just recently used an ex vivo porcine model to demonstrate the existence of reflex co-activation of the internal part of the TA muscle and CT muscle in the absence of central control [21]. The aim of this study was to find evidence of the morphology underlying the reflex response using histology, multiple immunofluorescence with synaptophysin/neurofilament (SYN/NF) and choline acetyl transferase/neurofilament (ChAT/NF), as well as transmission electron microscopy in human laryngeal tissue.

## 2. Results

### 2.1. Arrangement and Histological Characteristics of the Laryngeal Muscle Fibers Within the TA Muscle

**Figure 2:**
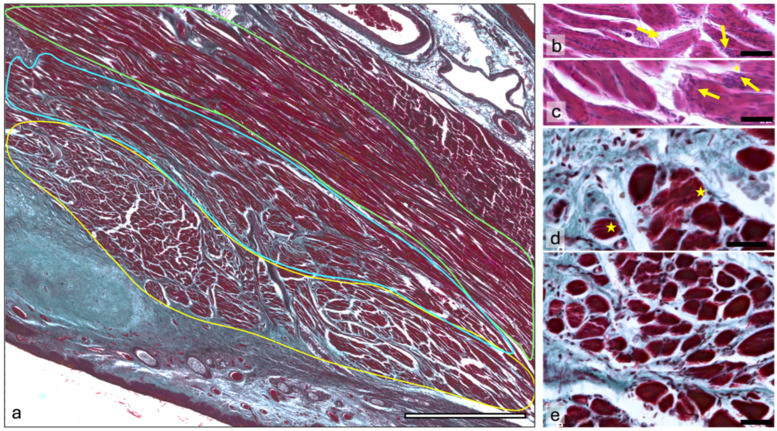
Histologic characteristics of the TA. Image (**a**) provides an overview of the TA muscle, stained with Goldner trichrome. The green outline marks the external part of the TA, the blue outline marks the intermediate area, and the yellow outline marks the internal part of the TA. A decrease in fiber size and an increase in the connective tissue between the muscle fibers can be observed. Images (**b**,**c**) show the HE staining of the internal part of the TA. Yellow arrows show terminal ends with a loose myofibrillar structure and nuclear concentrations. Image (**d**) is a coronal section of the internal part of the TA stained with Goldner trichrome. The yellow stars mark fibers with varying myofibrillar density. Image (**e**) represents an overview of the same area as in Image (**d**), stained with Goldner trichrome. Scalebar (**a**) = 2000 μm; (**b**–**e**) = 50 μm.

The muscle fibers within the TA muscle can be histologically grouped into three main areas. The first group, which is the most lateral compared to the vocal ligament, forms the outer part of the TA muscle (pars muscularis). Its fibers are mostly parallel and extend from the arytenoid cartilage to the thyroid cartilage. These fibers are densely packed, with some branching. They have a clearly visible striation and a uniform structure with relatively small differences in thickness. All fibers in this group have an oblique course and follow the shape of the thyroid cartilage toward a small insertion area near the anterior commissure (Figure 2a).

The intermediate area contains parallel muscle fibers that are organized in bundles and integrated into a coarse connective tissue network. The muscle fibers are more branched and of different sizes. Different directions of the bundles can be observed, indicating that this area belongs to the internal part of the TA muscle (vocalis muscle), where the fibers classically attach to the vocal ligament and originate from either the thyroid cartilage or the arytenoid cartilage (Figure 2a).

The innermost area next to the vocal ligament consists of numerous small muscle bundles that are enveloped by a honeycomb-like connective tissue network that originates from the vocal ligament. These muscle fibers are oriented differently and show the intertwining of fibers that originate from either the thyroid cartilage or the arytenoid cartilage. The size of the fibers is highly variable, and they show common branching (Figure 2a). The branches fan out toward neighboring muscle fibers and form tight junctions. Some muscle fibers can be seen in the connective tissue around the bundles. These disparate muscle cells are thin, mostly distributed longitudinally along the connective tissue, and appear to terminate in it. At the ends within the connective tissue, the muscle fibers in this area have less dense myofibril groups that end at the sarcolemma and do not run parallel to each other. The disparate muscle cells in this area have a much looser myofibril structure at their ends, with fewer myofibrils and concentrated nuclei (Figure 2b,c).

When looking at coronal (frontal) sections through the vocal folds, the structure of the musculature described above is not immediately recognizable. However, one can observe a decrease in the size of the muscle groups from the lateral to the medial side of the vocal fold as the groups move closer to the vocal ligament. Consequently, the amount of connective tissue near the vocal fold increases. Near the vocal fold, the muscle fibers are cut at an angle and fewer transverse fibers are visible. This reinforces the assumption that the muscle fibers attach to the vocal ligament and the associated connective tissue. A different density of myofibrils can also be observed in the transversely cut muscle fibers (Figure 2d). Lines of lower density on the muscle fibers indicate branching within the myofilament bundles (Figure 2e).

### 2.2. Ultrastructural Characteristics of Laryngeal Muscle Fibers

**Figure 3:**
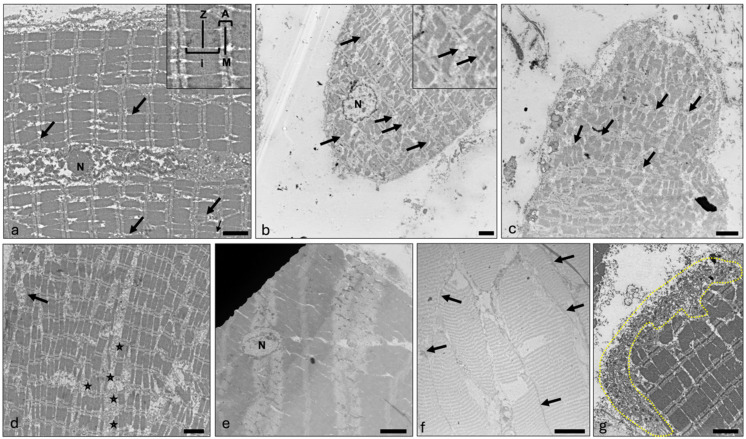
Ultrastructural characteristics of the vocalis muscle. Image (**a**) is an ultra-micrograph of a muscular fiber within the inner part of the TA. A and I bands as well as the M and Z lines are marked (magnification). A central nucleus (N) is visible. Black arrows mark spots where myofibrils ramify. Image (**b**) is an ultra-micrograph of a different muscular fiber. A nucleus (N) is visible near the sarcolemma. Branched myofibrils are more common (black arrows). Image (**c**) is an ultra-micrograph of the end region of another muscle fiber. Here, too, a myofibrillar branching can be seen near the cell membrane (black arrows). Image (**d**) is an ultra-micrograph of another muscle fiber. Several concentrated mitochondria (black stars) can be seen, while no triad organization is visible. Image (**e**) shows another muscle fiber with a centrally located nucleus (N). Image (**f**) is an ultra-micrograph of several densely packed fibers. The black arrows show direct connections between the muscle fibers with electron-dense plates at the junctions. Image (**g**) shows the end part of the muscle fiber. A large concentration of mitochondria is highlighted by the yellow dotted line. The organization is again irregular, with no visible dyad or triad organization. Scalebar (**a**–**g**) = 2000 nm.

The myofibrils of the larynx have the most important characteristics of the myofibrils of skeletal muscle. The A and I bands are clearly visible, as are the M and Z lines. However, one can also see myofibrils that show branching (Figure 3a), especially at the ends of the muscle fibers (Figure 3b,c). In these end regions, the myofibril bundles have a variable direction and stronger branching, with individual myofibrils ending at the cell membrane (Figure 3b). This aspect is consistent with the histologic findings. There are many mitochondria of different sizes (Figure 3d). The L-tubules are uniform; the T-tubules, however, can sometimes be seen in the periphery without a uniform triad organization (Figure 3d). The cell nuclei are mostly located in the periphery, but small nuclei can also be observed within the muscle fibers (Figure 3a,e). In areas with higher muscle fiber density and smaller muscle fibers, electron-dense connections between muscle fibers are present (Figure 3f). At the same time, in some muscle fibers, there are large cytoplasmic areas without myofibrils, in which cell nuclei or many mitochondria can be seen (Figure 3g).

### 2.3. Disposition and Properties of the Laryngeal Nerve Fibers

**Figure 4:**
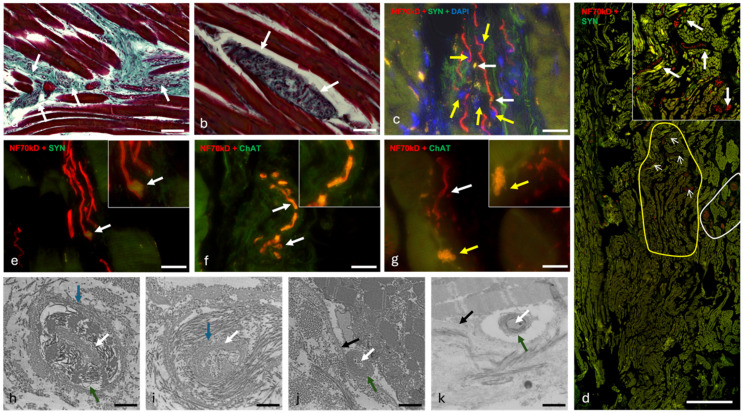
Examples of laryngeal nerves. Image (**a**) shows an area of the internal part of the TA muscle stained with Goldner trichrome. Multiple nerve bundles (white arrows) can be observed in connective tissue between muscle fibers. Image (**b**) is a slide from the external part of the TA muscle stained with Goldner trichrome. White arrows show a ganglion-like structure between the long muscular fibers. Image (**c**) is an immunofluorescence slide showing an overlay (NF + Syn + DAPI) of multiple nervous fibers of one nerve (central). Along the axons (red), vesicles (white arrows) are visible, and yellow arrows show the nuclei of Schwann cells. Image (**d**) is an overlay (NF + Syn) overview of the entirety of a TA muscle. The hilum-like area is outlined in white, and multiple neuron bundles (white arrows) are visible in the intermediate area of the TA (outlined in yellow). Image (**e**) is an overlay (NF + Syn). A nerve is in the center, and the white arrow marks a large syn-reactive bud within the nerve (white arrow). Image (**f**) is an overlay (NF + ChAT) showing the colocalization of ChAT vesicles along a nerve fiber (white arrows). Image (**g**) is an overlay (NF + ChAT) showing the presence of an axon (white arrow) without significant ChAT signaling, leading to a cholinergic synaptic bud (yellow arrow). Image (**h**) is an ultra-micrograph of a nerve fiber in an oblique section. The white arrow marks the axon, the green arrow shows the presence of a myelin sheath, and the blue arrow marks the endoneurium. Image (**i**) is an ultra-micrograph of non-myelinated axon. The white arrow marks the axon and the blue one the endoneurium. Images (**j**,**k**) show two myelinated axons (white arrow axon, green arrow myelin sheath) within the bounds of the sarcolemma (black arrow, Image (**j**)) or next to it (Image (**k**)). Scalebar (**a**,**b**) = 100 μm; (**c**,**e**–**g**) = 20 μm; (**d**) = 1000 μm; (**h**–**k**) = 1000 nm.

Nerve fibers (as seen in histological and immunofluorescence overviews) are abundant in the TA muscle and have a wide distribution within the muscle. They are present in all connective tissue sections of the aforementioned honeycomb structure (Figure 4a). Nervous structures can also be detected histologically between the long muscle fibers in the outer part of the TA muscle (Figure 4b). Larger nerve bundles are concentrated in the lateral part of the TA muscle, in the middle one-third of the antero-posterior length of the vocal cord. This zone functions as a hilus with concentrated neural and vascular structures (Figure 4d). The intermediate zone described above has several large groups of nerve fibers whose branches become increasingly frequent (Figure 4d). In contrast, the innermost region shows a broad distribution of small nerve fibers. These are not concentrated in terminal areas (Figure 4d).

Immunohistochemical detection of the nerve fiber bundles shows clear labeling of the nerve fibers with neurofilament 70 kD (NF) and numerous Schwann cell nuclei in the perineurium (Figure 4c yellow arrows). Additional labeling with synaptophysin (SYN) highlights some vesicles within the nerve groups (Figure 4c,e white arrows), some of them being of considerable size (Figure 4e). With the second antibody, choline acetyl transferase (ChAT), no such groupings are detectable (Figure 4f). With this antibody against ChAT, weak reactivity is observed in most nerve fibers, indicating the presence of ChAT vesicles along the axons (Figure 4f). Some axons do not exhibit ChAT vesicles along their course, especially when near a muscular fiber (Figure 4g).

Ultrastructural analysis shows nerves consisting of a combination of myelinated (Figure 4h) and unmyelinated fibers (Figure 4i). In some cases, individual nerve fibers can be observed that appear to lie within the cell boundary of the muscle fibers (Figure 4j) or directly next to it (Figure 4k).

### 2.4. Muscle Spindles Within the TA Muscle

**Figure 5:**
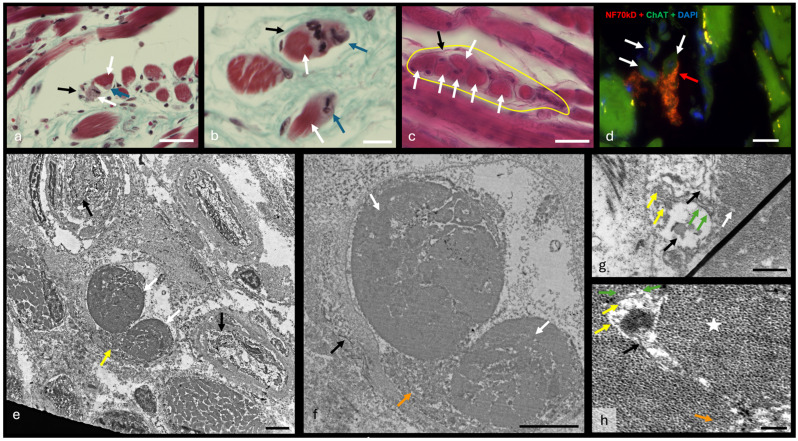
Laryngeal muscle spindles. Image (**a**) is a slice colored in Goldner trichrome detailing a muscular spindle. The white arrows mark intrafusal fibers, the blue arrows show synaptic terminations on said fibers, and the black arrow marks the capsule of the spindle. Image (**b**) is another slice in the same coloration, where multiple intrafusal fibers are seen (white arrow) with their corresponding neural termination (blue arrow). The black arrow marks a capsular layer, but no encompassing capsule is visible. Image (**c**) is a slice colored in HE showing a large muscular spindle (yellow outline) with the external capsule visible (black arrow) and multiple intrafusal fibers (white arrows). Image (**d**) is an overlay slice (NF + ACh + DAPI) showing a muscular spindle. No capsule is observable. The intrafusal fibers (white arrows), some with multiple nuclei, are visible. A large neural endplate is marked by the red arrow. Image (**e**) is an overview ultra-micrograph showing a central muscle spindle with two intrafusal fibers (white arrows). Dark arrows mark two axons near the synaptic area, and a large synaptic terminal (yellow arrow) is visible next to the intrafusal fibers. Image (**f**) is a detail of Image (**e**) with two intrafusal fibers (white arrows), an axon (blue arrow), and the synaptic terminal (orange arrow). Image (**g**) shows a section of the sensory terminals on the surface of an intrafusal muscle cell. The two terminal buds (dark arrows) contain several vesicles (yellow arrows). The synaptic density is visible at the tip of the green arrows. Image (**h**) shows a sensory terminal penetrating deep into the sarcoplasm of an intrafusal cell (white star). The sarcolemma is marked by the orange arrow. We can recognize the axon (dark arrow), synaptic vesicles (yellow arrows), and synaptic density (green arrow). Scalebar (**a**,**c**) = 50 μm; (**b**) = 30 μm; (**d**) = 20 μm; (**e**,**f**) = 2500 nm; (**g**) = 500 nm; (**h**) = 200 nm.

Histologically, spindle-like structures are found in the entire pars interna of the TA muscle (Figure 5a–c). These are small muscle fibers with neural endings that do not have a clear outer capsule (Figure 5a,b). The inner capsule is visible (Figure 5a,b). Immunofluorescence shows groups of intrafusal muscle fibers together with accompanying cholinergic nerve fibers, but no clear outer wall is visible (Figure 5d). Of interest in this case is the presence of a neuronal connection between these muscle fibers and an extrafusal muscle fiber nearby (Figure 5d).

Ultrastructurally, muscle spindles are visible in the pars interna of the TA muscle (Figure 5e). Groups of intrafusal cells are accompanied by myelinated nerve fibers (Figure 5e). An outer capsule is not recognizable (Figure 5e). The inner capsules and the associated nerves are surrounded by a dense extracellular collagen network (Figure 5f). The diameter of the intrafusal fibers ranges from about 15 µm to less than 1 µm. Intrafusal fibers have a distinct sarcoplasmic reticulum and numerous mitochondria. Intrafusal muscle fibers have small sensitive neuronal endings with a diameter between 250 nm and 1 µm, which are distributed both on the surface of the muscle fibers (Figure 5g) and deep in the sarcoplasm (Figure 5h).

### 2.5. Distribution and General Characteristics of Laryngeal Neuromuscular Synapses

**Figure 6:**
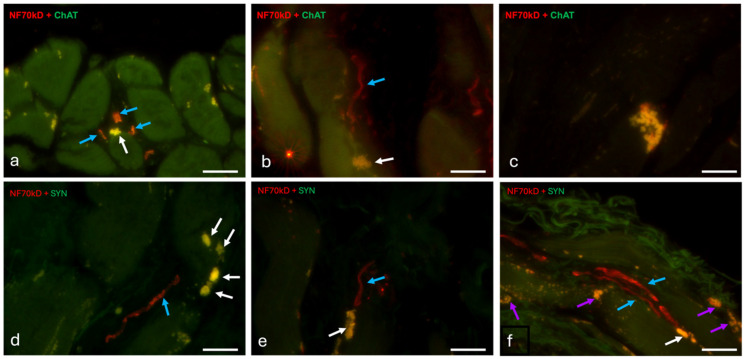
Neurofilament-acetylcholine immunofluorescence of laryngeal synapses. All images in the series are taken at 100× magnification. Images (**a**–**c**) are dual-stained neurofilament (NF)/choline acetyl transferase (ChAT) sections, while images (**d**–**f**) are dual-stained neurofilament (NF)/synaptophysin (SYN) sections. Image (**a**) demonstrates a terminal bud (white arrow) surrounded by several neural terminals (blue arrows) that are more intensely stained with NF, although some ChAT signal is also present. In Image (**b**), we again see a typical terminal bud (white arrow), but only one axon is approaching it (blue arrow). Image (**c**) shows an elongated termination covering the entire terminal region of a muscle fiber. Image (**d**) shows several terminal buds (white arrows) originating from the same neuron (blue arrow marks the axon). Image (**e**) shows an example of a larger monolithic terminal bud (white arrow) and the associated axon (blue arrow), and Image (**f**) shows two axons (blue arrows) approaching the same endplate (white arrow). Several other endplates can be seen in the surrounding muscle fibers (purple arrows). Scalebar (**a**–**f**) = 20 μm.

Immunohistochemical detection of synapses shows an extensive distribution within the TA muscle, without a clear organization toward terminal insertions into the adjacent connective tissue. The synaptic terminals vary in size and branching, with simple synaptic terminals having a diameter of 5–25 µm (Figure 6a–c). Their shape varies, with bud-like endings being common (Figure 6a,b), but more elongated plaque endings also occur (Figure 6c). These monolithic neuromuscular junctions are observable in the case of cholinergic synapses. When looking at the antibody responses against synaptophysin and neurofilament, further ramifications of the synaptic terminals are evident. Some muscle fibers show smaller neuronal endings distributed over a larger area and consisting of buds with a diameter of 5–8 µm (Figure 6d). However, more compact synapses with larger buds (27 µm) are also recognizable (Figure 6e). Several neurons are visible interacting with the same muscle fiber (Figure 6f). The role of such a connection is uncertain, as usually one of the neurons appears to terminate in a motor synapse, while the other nerve fibers do not appear to have efferent synaptic terminals (Figure 6f).

**Figure 7:**
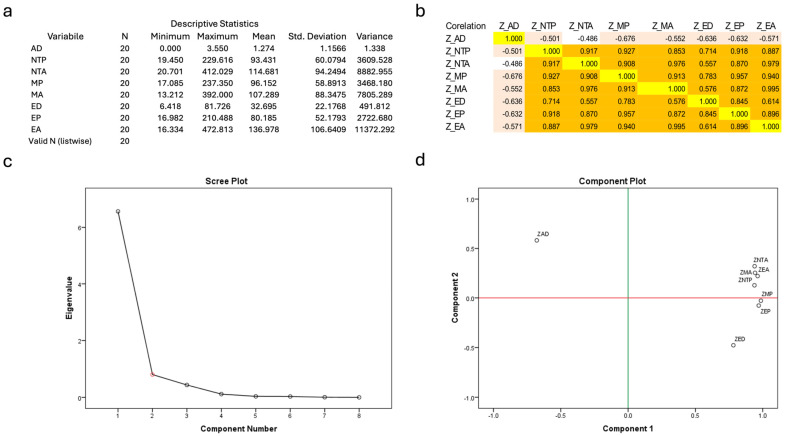
A statistical analysis of the synapses identified with the aid of immunofluorescence. AD = axonal diameter, NTP = perimeter of the neural terminal, NTA = area of the neural terminal, MP = perimeter of the muscle-touching endplate, MA = area of the muscle-touching endplate, ED = diameter of the synaptic endplate, EP = perimeter of the synaptic endplate, EA = area of the synaptic endplate. Table (**a**) shows the main descriptive statistical parameters of all parameters recorded for the 20 synapses analyzed in this way. It can be seen that the variance is generally higher for the area parameters, indicating that there is a high degree of local variability between the laryngeal synapses. It can also be seen that the area of the endplate is larger than the average area of the neuronal terminals, indicating presynaptic folding. In table (**b**), one can see the correlation matrix between the parameters. The correlation between the parameters is either high or very high (0.7–1, orange background), with only the axonal diameter showing a moderate correlation (pink background) with the other parameters (0.5–0.7), which is to be expected. The white background marks low correlation and the yellow background marks perfect correlation. Image (**c**) shows the individual values for each parameter and demonstrates that the variance between the features of the synapses (2–8 on the x-axis) is much more significant than for the axonal diameter (value 1). Image (**d**) shows the two-factorial reduction in the component matrix, where the first component (z-axis) evaluates the synaptic elements, and the y-axis evaluates the presynaptic features (the axonal diameter was the only relevant parameter). Thus, based on the concentration of synaptic parameters measured, it can be seen that synaptic measurements can be simplified to the overlap area (EA—endplate area) between two markers for subsequent analyses, opening the way for a more comprehensive evaluation of synapses in the laryngeal region using only overlap data.

When analyzing the numerical data extracted using the tools described previously, we can observe a large variability between synaptic parameters (Figure 7a), which is highly pronounced for the area parameters (NTA = neural termination area, MA = muscle-contacting endplate area, EA = active endplate area). In comparison, the axonal diameter shows a relatively low variance, indicating that the increase in nerve fiber size is generally not strongly correlated with synapse size (r = 0.486–0.676, Figure 7b), while all other parameters are relatively strongly or very strongly correlated (Figure 7b). Of these endplate parameters, the endplate diameter shows the weakest correlation with the others (r = 0.557–0.957). Using the Bartlett test for sphericity, we obtained a chi-squared value of 307.675, which shows a high degree of dependence between endplate values (sig. 0.0001 < 0.05), indicating that although the individual correlation of some parameters of the synapse (such as endplate diameter) is not as strong, the overall predictive value is similar to the other size parameters. The intrinsic values for the total variance are shown in Figure 7c, which allows us to divide the variables into two groups, one with the axon diameter (position 1) and one with the endplate parameters (2–8). Of these parameters, Figure 7d shows that the muscle-contacting endplate perimeter (MP) and the endplate perimeter (EP) are the least influenced by axon size and can, therefore, be used as reliable indicators of the size of a synapse. Since the endplate perimeter defines the area where vesicular markers (SYN, ChAT) and neuronal markers (NF) overlap, we propose the use of the endplate perimeter to assess synapses in larger datasets. In our dataset, when comparing this parameter between synapses labeled with ChAT and synapses labeled with SYN, we found that cholinergic synapses are generally smaller than the general pool of synapses observed by SYN staining (one-tailed p1 = 0.032 p2 = 0.037).

### 2.6. Ultrastructural Characteristics of Laryngeal Synapses

The neuromuscular connections of the larynx are numerous and do not follow a rigid arrangement pattern, as they are found in preparations from the pars interna of the TA muscle (vocalis muscle). The observed synaptic endings can be morphologically categorized into three main subtypes.

**Figure 8:**
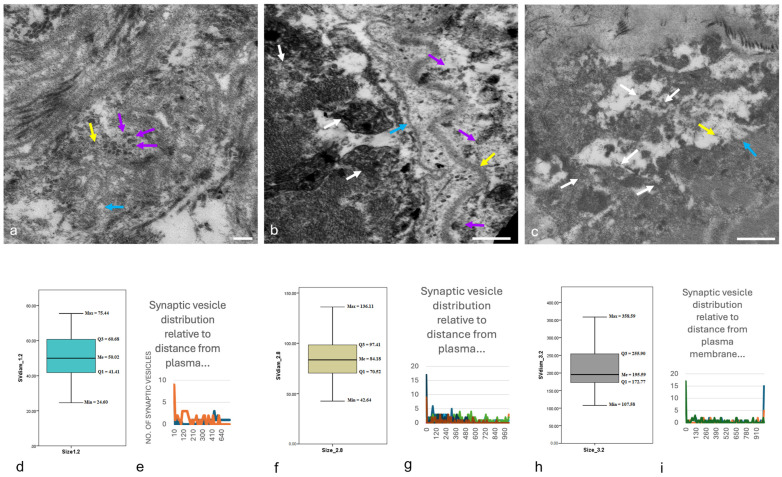
Ultrastructural features of the neuromuscular synapses of the larynx. Image (**a**) demonstrates a type 1 synapse (normal neuromuscular junction). One can observe deep folding in the postsynaptic membrane (blue arrow) and some folding in the presynaptic membrane (yellow arrow). Several vesicles are visible (purple arrows). Image (**d**) shows the size range of these vesicles, while Image (**e**) reveals their relative distribution to the plasma membrane. Image (**b**) shows a type 2 synapse, where one can observe some folding in the postsynaptic membrane (blue arrow) and deeper folding in the presynaptic membrane (yellow arrow). Several vesicles can be seen (purple arrows) that are larger than in Image (**a**). Mitochondria (white arrows) are concentrated near the postsynaptic membrane. Image (**f**) shows the size range of these vesicles, while Image (**g**) shows their relative distribution to the plasma membrane. Image (**c**) shows a type 3 synapse (which is considered sensitive in nature). Very little folding can be observed in both the postsynaptic membrane (blue arrow) and the presynaptic membrane (yellow arrow). The synaptic cleft is very narrow and cannot be distinguished in most cases. Vesicles (white arrows) are visible on both sides of the membrane. Graphic (**h**) shows the size range of these vesicles, while Graphic (**i**) illustrates their relative distribution to the plasma membrane. Scalebar (**a**) = 2500 nm; (**b**) = 500 nm; (**c**) = 1000 nm.

The first subtype consists of classic neuromuscular junctions with strong folding of the junctions (Figure 8a). These show the classic characteristics of a neuromuscular junction. The main axon is located near the cell wall of a muscle fiber, partly accompanied by a Schwann cell. Around this, several terminal buds with a diameter of 1–2 µm are inserted into deep furrows of the sarcolemma or can be seen in the sarcoplasm. These buds have an electron-dense, thick basement membrane and several synaptic vesicles next to or in contact with the basement membrane (Figure 8a). Within these buds, electron-dense neurofilaments and neurotubules are occasionally seen. Remarkably, postsynaptic folding occurs but is not complex (Figure 8a). Associated presynaptic folding, on the other hand, occurs regularly (Figure 8a). The postsynaptic cytoplasmic region contains numerous mitochondria and a distinct sarcoplasmic reticulum. The synaptic cleft is 70 nm wide (SD 24.8 nm). In type 1 synapses, the average number of vesicles was 46 (SD 8.4), the size of which is shown in Figure 8d. The distribution of vesicles was concentrated near the plasmatic membrane with a relatively stable decrease in vesicles toward the interior of the terminal bud (Figure 8e).

The second subtype is the synapse most frequently found in the samples analyzed. It consists of larger neural endings with a diameter of 2–5 µm, which are located near the sarcolemma in shallow depressions or in the sarcoplasm. They occur either as single endings or in complex synapses consisting of several synaptic endings. Occasionally, Schwann cells are found that surround the endings. The membrane layer is electron dense and thick on both synaptic sides (Figure 8b). The synaptic cleft is up to 350 nm wide at its widest points (avg. 181.91 nm sd 48.93 nm) and is usually more electron dense than the surrounding cytoplasm or the extracellular space. Within the synaptic cleft, electron-dense vesicles with a diameter of 40–800 nm can be seen (Figure 8b). Discrete pre- and postsynaptic folds can be observed (Figure 8b). The cytoplasm within the terminal buds is densely packed with synaptic vesicles, especially in the vicinity of the synaptic cleft (Figure 8b). Rough endoplasmatic reticulum and mitochondria are differently pronounced. Some buds have neurotubules (Figure 8b) or myelinated branches of the axon. The postsynaptic cytoplasm is rich in mitochondria and sparse in myofilaments (Figure 8b). Muscle fiber nuclei can be seen near the synapse. In type 2 synapses, the average number of vesicles was 60 (SD 10.6), the size of which is shown in Figure 8f. The distribution of vesicles was concentrated near the plasmatic membrane and showed a more abrupt difference from the number of bound vesicles (0 nm from the membrane) to a large concentration near the membrane (10–500 nm) and a more pronounced decrease thereafter (Figure 8g).

The third type of synapse (Figure 8c) consists of larger terminals with several large synaptic buds of 5–10 µm in length, which are located in indentations of the sarcolemma or on the surface of the muscle cell (Figure 8c). The synaptic cleft is narrow, 20–30 nm wide (Figure 8c), and the synaptic membranes can be seen as a common, electron-dense layer. Synaptic folding is present but not complex (Figure 8c). The axoplasm within the terminal buds is not dense and shows only sparse vesicles and neurofilaments (Figure 8c). The postsynaptic sarcoplasm is densely packed with mitochondria and small vesicles that are in contact with the synaptic membrane. For type 3 synapses, only the vesicles identified within the neuronal membrane were counted. The average number of vesicles was 50 (SD 12.66), the size of which is shown in Figure 8h. While the number is higher than in type 1 synapses, their concentration was visually lower, which is reflected in the larger overall size of the buds. The vesicles were concentrated in two peaks, one near the plasmatic membrane and the other in the center of the buds, with a relatively constant distribution in between (Figure 8i).

A total of 15 synapses were manually identified and scored using the numerical methods described, including 2 type 1 synapses, 10 type 2 synapses, and 3 type 3 synapses, as defined above. Synaptic vesicles were identified in varying numbers in all cases, ranging from 25 to 119 vesicles per endplate (mean 54.88, SD 26.65727469).

### 2.7. Neurons Within the TA Muscle

**Figure 9:**
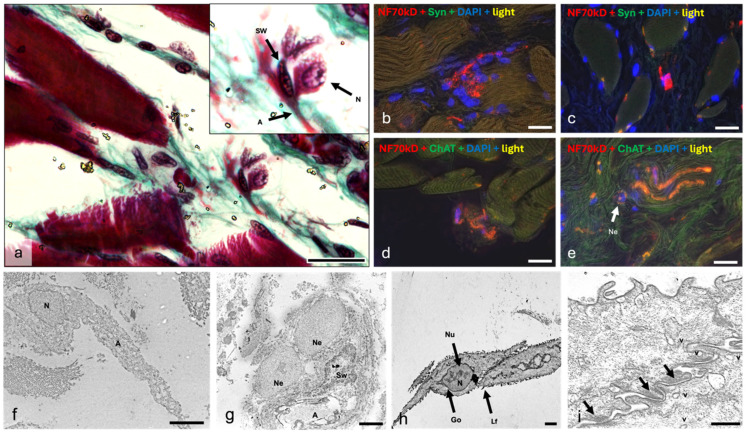
Possible neurons within the laryngeal muscles. Image (**a**) is a slide stained in Goldner trichrome showing a pseudo-unipolar neuron between muscular fibers. N = neuron nucleus, A = axon, SW = Schwann cell, Ne = neuron. Images (**b**–**e**) are overlay immunofluorescence slides showing singular neurons in the vicinity of muscle cells (**b**,**c**,**e**) or nerves (**d**) with synapses being visible in the proximal muscular cells in images (**c**,**d**). Image (**f**) is an ultra-micrograph showing a unipolar neuron in connective tissue between muscular fibers. It presents a large triangular body, a single central nucleus (N), and an axon (A). Image (**g**) contains two large uninuclear round cells (presumed to be neurons—Ne) located within the endoneurium of a myelinated nerve fiber (A), together with a Schwann cell (Sw). Image (**h**) contains two bipolar neurons, with Image (**i**) being a detail thereof. We can observe the central nucleus (N) with a nucleolus (Nu), a Golgi apparatus (Go), and a lipofuscin inclusion (**f**,**i**). The two possible neurons synapse in an area detailed in Image (**i**). Synaptic densities are visible on either side of the membrane (black arrows) as well as vesicles (arrowheads). Synaptic folding is present. Scalebar (**a**–**e**) = 20 μm; (**f**–**h**) = 2500 nm; (**i**) = 500 nm.

Occasionally, cells with the characteristics of neurons (Figure 9a) are visible in the inner part of the TA muscle (vocalis muscle) on histological sections. These are round or triangular, between 8 and 11 µm long, and have a large central nucleus with a single nucleolus and little cytoplasm. The cells have reddish-pink (in Goldner’s trichrome) projections that are either bipolar or pseudounipolar with a diameter of 2.2 µm, consistent with the axons measured in the immunofluorescence study (Figure 9a). Schwann cell nuclei occur in the vicinity of the neurons (Figure 9a). These cells are located either in the vicinity of muscle cells (Figure 9a) or in the vicinity of larger neuronal structures.

Cells with such characteristics are also visible by immunohistochemistry (Figure 9b–d). Most of them are located in the immediate vicinity of the muscle fibers (Figure 9b–d), although one such cell was also observed in the vicinity of a larger nerve (Figure 9e). The cells in this case are either round (Figure 9b,c,e) or oval (Figure 9d) with a size of 10 µm for the round cells and 15 × 9 µm for the oval cells. All have a large central nucleus that reacts with DAPI. Cellular projections were observed in 3 of the 4 cells encountered (Figure 9b–d), with one cell cut in the transverse direction (Figure 9e). The projections are reactive to neurofilament in all cases (Figure 9b–d) and are also labeled with the antibody against choline acetyl transferase (Figure 9d,e). In the case of Figure 9d, the cell appeared to be cut in the transverse direction. The peripheral signal highlights synaptic vesicles, as it responds more strongly to choline acetyl transferase (ChAT), while the central signal responds more strongly to neurofilament (NF) (Figure 9e). In cases where such cells lie between muscle fibers (Figure 9b–d), synaptic endings can be observed nearby.

Ultrastructurally, cellular structures resembling neurons can be seen between the laryngeal muscles (Figure 9f–h). These cells generally have a large solitary nucleus with one or no visible nucleolus. Their size is between 5 and 16 µm, depending on the measured diameter. Their shape varies from fusiform (Figure 9h) to triangular (Figure 9f), to almost round (Figure 9g). The cytoplasmic projections are usually bipolar, although some unipolar cells are also visible (Figure 9f). Some of the cells encountered were visible in contact with Schwann cells (Figure 9g). The cells observed (Figure 9h) have numerous fibrillar structures in the cytoplasm (neurofilaments), lipofuscin inclusions (Figure 9h), mitochondria, and a perinuclear Golgi apparatus. There are also a number of apparent synapses between the two cells shown in Figure 9i. The synaptic cleft is 20–25 nm wide, and distinct pre- and postsynaptic densities are recognizable. Some synaptic vesicles are present on both sides of the synapse (Figure 9i).

## 3. Discussion

The unique function of the vocal folds in the human body is supported by specialized muscular and neural structures that are not yet fully understood. This is not surprising given that other fast-twitch muscles with constant activity have adapted structures and gene expressions with respect to their neuromuscular apparatus [22], and the arrangement of muscle fibers reflects this. The results we have obtained here, as well as the findings of previous authors, show that the muscle fibers in the internal part of the TA muscle (vocalis muscle—VM) do not run in an anterior–posterior direction in line with the vocal ligament [6,23,24,25], confirming that the contraction of the VM both shortens and tenses the vocal fold. However, the apparent histological differentiation into three morphological zones is a new concept. We assign the first group described to the outer part of the TA muscle (pars muscularis) and the other two groups to the internal part of the TA muscle (vocalis muscle—VM). The distinction between the second and third groups is essential due to their interaction with the surrounding connective tissue. The relationship of the third group to the honeycomb-like connective tissue cells allows this area to act as an isotropic tissue during vocal vibrations. This was demonstrated in a recent functional study on a porcine larynx [8] in which muscle tissue compression was restricted to this area. Consequently, any contraction of the muscle fibers in the third group tends to control the elastic properties of the oscillatory system. A large proportion of these fibers enter the composition of the second group for most of their length, as they follow an oblique pattern of progression, as described in a previous study [11]. Thus, the role of the second group is to provide a directional impulse to the third group, which is more in line with the classical role of the vocalis muscle. In turn, the lower density of distal myofibrils and the higher number of branches in group 3 serve to distribute the forces exerted by the oscillatory waves more evenly in this region, favoring isotropic conditions. Apart from the cellular ramifications, the laryngeal fibers of the vocalis muscle have ultrastructural features that distinguish them from the skeletal muscles in other regions.

Like skeletal muscle, the cells are generally large and multinucleated, with the nuclei located in the periphery [24,25], but occasionally, centrally located nuclei are also found. However, myofibrillar branching is not found in skeletal muscle. The mitochondria between the myofibrils and at the cytoplasm-rich cell ends are more comparable to cardiac muscle [26]. The absence of numerous triads is also similar to cardiac muscle cells, as is the presence of intermuscular junctions [27]. These junctions are electron dense and arranged transversely, suggesting a role in ion transfer. All these features lead to the assumption that the internal part of the TA muscle (vocalis muscle) contains a unique (specialized) form of striated muscle fibers. It is for this reason that we propose the term “myolaryngovocalis” to refer to this type of muscle fiber henceforth.

The wide distribution of nerve structures in the larynx indicates the presence of a complex neuromuscular system at this level. The multiple innervation of laryngeal muscle fibers is well documented [9,15]. These nerve endings are mostly located in the middle third of the vocal folds in an antero–posterior direction [17], which explains the presence of the hilus-like structure described above in this area. We only show the colocalization of ChAT within the nerve bundles in the present study, but other experiments have shown the presence of other non-cholinergic nerve fibers with different reactivities [16]. This would explain the smaller non-myelinated nerve fibers that we observed ultrastructurally.

The presence of muscle spindles in the larynx is still a controversial topic among experts, with evidence being presented on both sides. On the one hand, numerous authors have described the presence of muscle spindles in human TA muscle [6,9,28,29,30,31]. On the other hand, recent studies using specific antibody labeling of myosin heavy chains show the absence of intrafusal myosin types [19], and serial analysis of larynxes across multiple ages has revealed no muscle spindles [32]. In our study, spindle-like structures were detectable in the internal part of the human TA muscle (vocalis muscle), mainly located in its inner part. This corresponds to the arrangement described by other authors [28]. However, with the methods we used here, we could not clearly identify an outer capsule of these spindle-like structures. Some spindles, such as those highlighted by immunofluorescence labeling, did not show nuclear sac fibers. An aspect that has been described in the literature [6,33], neural structures are visible in association with the intrafusal nerves, which vary in diameter and are either myelinated or unmyelinated, similar to other spindles described in the literature [34]. However, the entirety of nerves approaching the muscle spindle could only be observed on histologic sections. Intrafusal nerve fibers show increased mitochondrial representation (as described in [34]) within the muscle spindle, and branching is evident. The intrafusal fibers had a small diameter of 5–8 µm, smaller than fibers described in other intrinsic laryngeal muscles [35].

When looking at the neuromuscular synapses of the larynx, their wide distribution over the TA muscle (vocalis muscle) is particularly striking. This can be easily observed in overview images of immunofluorescence-labeled samples. The distribution is different from other skeletal muscles, with the exception of the extraocular muscles, which show a similar fast-twitch, precise motility [36]. The reasons for such a distribution may be as follows in the internal part of the TA muscle (vocalis muscle): on the one hand, the observed muscular arrangement means that most muscle fibers within the vocalis muscle attach to the vocal cord, especially in the middle third of the vocal fold, when looking antero-posteriorly. This explains why most of the endplates are concentrated here, both in our study and in other studies [9,17]. The second mechanism that favors the accumulation of synapses in this area has to do with reinnervation patterns. It has been observed that reinnervation originates from nearby neural terminals [37] leading to large, multifiber endplates. The vocalis muscle area is most active during phonation [38] and is subjected to repeated microtraumas and multidirectional stresses during glottal closure when the two vocal cords come into contact. This, in turn, would lead to damage to this area over time and the need for reinnervation. Goerttler pointed out that the intertwining of muscle fibers can also occur in other skeletal muscles when fibers are damaged and new fibers are inserted transversely [6].

The different typology of the laryngeal synapses illustrates the complexity of the neuromuscular apparatus at this level. Large en plaque terminals appear to be cholinergic motor endplates that react immunohistochemically with antibodies against acetylcholine and neurofilament. They correspond to the first ultrastructural subtype mentioned above. They are also present in other skeletal muscles but are rather sporadic in the present study, both immunohistochemically and ultrastructurally. Where they are present, the functional folding is not as pronounced as in other skeletal muscles [26]. Since functional folding is generally proportional to signal amplification [39], laryngeal synapses appear to have adapted to the smaller size of the motor unit and the need for finer and more diverse activation patterns. Less folding of the synapses ensures a more gradual activation of the TA muscle and possibly a shorter refractory period of the muscle. This is also confirmed by the number of vesicles encountered, which on average is the smallest of the three types, while their relative distribution (Figure 8f) indicates that only a gradual number of vesicles reach the membrane at any given time.

This is exacerbated by synapses of the second ultrastructural type. The large synaptic cleft and the large number of terminals could lead to a slow diffusion of the neurotransmitter, resulting in a sustained but gradual contraction. The larger average size of the vesicles could thus serve as a regulating factor through an increased release of neurotransmitters in the junctional fold. In addition, these synapses appear to be more active or more frequently activated, as shown by the increased presence of synaptic vesicles near the membrane. These connections resemble the synapse à distance [40] of smooth muscle and could fulfill a similar function, especially in conjunction with the observed intermuscular connections. Synapses consisting of multiple en plaque terminations that were reactive in the immunofluorescence analysis were present. Such terminations were observed in the extraocular muscles [41] that have a motor function. These correspond ultrastructurally to either the second or the third subtype described here. However, we hypothesize that the third subtype is sensitive due to the small junctional gap and the presence of vesicles on both sides of the synapse.

The final important finding of our study is the apparent presence of neuronal bodies in the larynx. Intramuscular ganglia containing either pseudo-unipolar or bipolar neurons have been described in the TA muscle (vocalis muscle) of dogs [16], but the neuronal structures observed in our studies cannot be considered part of ganglionic structures. Nevertheless, all observed neurons are either bipolar or pseudo-unipolar and have a uniform size of up to 11 µm. The cells are characterized by the presence of a single central nucleus and are identified as neurons by their reactivity with the antibodies used in immunofluorescence as well as by their nuclear proximity to other neuronal structures and apparent synaptic connectivity in electron microscopy. As pointed out by other authors, their role is controversial, with either a sensory or a vegetative role being discussed, as they are associated with non-cholinergic neurotransmission [16,20]. We believe that a sensory role is more likely, as they are closely associated with muscular and neural structures, with no such neurons observable along vascular or glandular structures in the samples we examined.

Our study is limited by the use of postmortem tissue from body donors, as conditions are not optimal for electron microscopy studies, which should preferably be taken from living tissue (surgical tissue) or immediately after death. However, few patients undergoing laryngeal surgery have pathologies that do not affect muscle tissue, and the protocols for body donation mean that tissue is only available between a couple of hours and two days after death. Nevertheless, we remain confident that we can extend future research in the directions mentioned.

## 4. Material and Methods

### 4.1. Subjects and Samples

The present study was performed on human tissue obtained from human cadavers donated by written testamentary disposition in the years 2021–2023 and in accordance with German law to the Institute of Functional and Clinical Anatomy of Friedrich-Alexander Universität Erlangen-Nürnberg (FAU) as well as to the Anatomical Institute of Christian-Albrechts-Univeristy (CAU) of Kiel, Germany. Human larynges (*n* = 5, aged 67–81, 2 male, 3 female) from the CAU Kiel were harvested already in the years 1990–1995, fixed in 4% formalin, and embedded in paraformaldehyde, and the larynges were cut as a whole in sagittal and frontal planes (7 µm thick sections). At the FAU, a total of 10 complete larynges from 10 different subjects (5 male, 5 female, aged between 55 and 86 years) were prepared for immunofluorescence and transmission electron microscopy (TEM). None of the body donors from the CAU or FAU had recent trauma, infections (including the donor site), or other known diseases that may have affected the donor sites in question. The tissue from the 10 larynges at the FAU was collected from the body donors within 24 h after death by qualified personnel of the institute. A specimen consisting of the larynx and the first 2 tracheal rings was obtained from each subject and then preserved by freezing at −20 °C. For further processing, the pieces were thawed and subsequently sagittalised. Microdissection using an OPMI Pentero surgical microscope (Carl Zeiss, Oberkochen, Germany) was performed on each half of the larynx, with one being processed for fluorescence microscopy and one for transmission electron microscopy.

The dissection was performed on one side of the larynx, and the entire vocal fold was dissected together with the arytenoid cartilage, which was used for orientation prior to paraffin embedding. The vocal fold was sharply separated from the inner wall of the thyroid cartilage using techniques described in anatomical manuals [42]. Thus, the dissection began with a sharp incision along the upper edge of the cricoid cartilage. Once the cartilage was exposed, the dissection was continued upward along the conus elasticus until the inner wall of the thyroid cartilage was reached. A sharp dissection was then performed from the cartilage until the vocal folds were exposed. The samples obtained were immediately placed in 4% PFA. In the half of the larynx intended for TEM, the mucosa was incised and removed from the thyroarytenoid muscle, and two small samples (with a maximum diameter of 2 mm) were taken from the area directly lateral to the vocal fold in the transverse plane (corresponding to the innermost part of the thyroarytenoid muscle) and fixed by immersion in a home-made ITO solution (paraformaldehyde 25%, glutardialdehyde 25%, cacodylate buffer 0.1 M, picric acid-saturated solution). Excess material was disposed of according to the standards of the Institute for Functional and Clinical Anatomy, FAU, Erlangen, Germany.

### 4.2. Histology

Histological slices were performed on larynges donated by written testamentary disposition and in accordance with German law to the Anatomical Institute of CAU Kiel, Germany as mentioned above. Sections that were 7 µm thick were alternated (every 2nd section) and stained with Goldner trichrome and with hematoxylin eosin as per techniques described in [43] (1 transverse and 1 coronal section). Each mounting contained the entirety of the larynx on the slice, allowing for an adequate overview.

### 4.3. Immunofluorescence Study

Neurofilament immune-coloring techniques are widely used to mark all neural components, being especially useful for marking axons [44,45], and have been a proven go-to for such purposes, being used also in experimental laryngeal studies [46,47]. This marker would be more concentrated in neural bodies and projections. Simultaneous markers for synaptic terminations were paired with this. Motoneurons are cholinergic, so for their identification, choline acetyl transferase-specific antibodies were used. Synaptophysin coloration was added to the experimental protocol because some authors described non-cholinergic neurons within the larynx [16,20]. Synaptophysin is proven to be reactive to all types of efferent or afferent neural terminations [48,49], thus permitting the identification of any type of synapse present within the laryngeal muscles.

For immunofluorescence, the vocal folds obtained from the dissection described above were further processed by removing the arytenoid cartilage. Then they were sectioned, with a microtome, in half, either lengthwise or transversely, to trim the size before paraffin embedding. Following this, the samples were embedded in paraffin, and slices 7 μm thick were made. The sections were then processed for double immunofluorescence (see Table 1) with either neurofilament—NF (mouse anti-NF 1:50 Biozol MUB 1303p diluted in tris-buffered saline tween—TBST + 2% BSA + 2% Triton X-100) and choline acetyl transferase (ChAT) (Goat anti-ChAT 1:50 Millipore AB 144p LOT: 2916187 ) or neurofilament—NF (mouse anti-NF 1:50 Biozol MUB 1303p diluted in TBST + 2% BSA + 2% Triton X-100) and synaptophysin—SYN (Synaptophysin conjugated with Alexa 488 Proteinthech CL488-67864 diluted in TBST 1:100). The secondary antibodies used were as follows: rabbit-anti-goat Alexa 488 1:1000 Invitrogen, goat-anti-mouse Alexa 555 1:700 Invitrogen, and DAPI 1:1000 Invitrogen diluted in TBST. All antibodies used, and their origin and controls, are given in Table 1.

### 4.4. Transmission Electron Microscopy

Samples obtained through the method described above were left for fixation in a refrigerator for at least 12 h. Secondary fixation was performed by soaking the pieces for 2 h using OsO4 and 0.1 M cacodylate buffer (Carl Roth GmbH, Karlsruhe, Germany) (1:1 solution). Afterward, the pieces were embedded in EPON (Carl Roth GmbH, Karlsruhe, Germany), and semi-thin 1 μm slices were cut with a Reichert Ultracut S microtome (Leica Microsystems, Wetzlar, Germany). These were stained with toluidine blue. Inspection areas were chosen as follows:

- Areas with a medium density of muscular fibers;
- Areas where other structures were visible, such as nerves and large vasculo-nervous groups together with the muscle.

This led to the best results for the targeted structures (nerves, synapses, muscle spindles). The marked areas were then cut out for ultra-thin 50 nm sections. These were cut with a diamond knife and mounted on pinhole copper grids (Science Services, Munich, Germany) with a large opening of 2 × 1 mm diameter, which were first covered with a Pioloform film (Plano, Wetzlar, Germany) made of a liquid phase (thickness = 2–3 nanometers). In some cases, 100-mesh copper grids from the same manufacturer were used. These were used uncoated and offered better stability for the ultrathin slices at the cost of losing access to any interesting structures located under the gridlines. All images were captured on a Zeiss Leo 906 E microscope (Carl Zeiss, Oberkochen, Germany) using a TRS 2048 (Tröndle Restlicht Verstärkersysteme, Moorenweis, Germany) digital camera system. Electron microscopy slices obtained as described presented good ultrastructural characteristics and were clear for images taken up to 100.000× magnification. Freezing preservation of the tissues was necessary because of the inconsistent nature of body donor organ availability and the predicted timeframe between prelevation and processing. This was deemed acceptable since a number of studies have explored the effects of slow freezing on ultrastructures and found that freezing can be a useful method of preservation with varying degrees of success [50,51,52]. The use of paraformaldehyde to fix such tissues is well established in the literature, and no significant degradation has been documented for TEM purposes [53]. Thus, a combination of paraformaldehyde and glutaraldehyde, as used here, has become the standard protocol for our institution and others [54]. In our experience, this does not significantly degrade the ultrastructure.

### 4.5. Data Analysis

The images obtained for both TEM and fluorescence microscopy were analyzed using the FIJI image processor (version 2.14.0) [55] to extract numerical measurement data. This took the form of manual measurements of relevant structures identified by visual features and the use of two macros described in the literature. For immunofluorescence, *n* = 20 visually identified synapses were processed with the macro aNMJ-morph, as described in the article by Minty et al. [56]. Segmentation was omitted as automatic segmentation has only been shown to be effective for mouse NMJs, which differ from those of humans as they are less complex. A total of 8 variables were determined for the analysis. Some synapses had no visible axon; in this case, the axonal parameter was assigned the value 0. The variables thus obtained were imported into SPSS version 29.0.2.0 (IBM, Armonk, NY, USA), and a principal component analysis was performed for the 8 variables. Principal component analysis is a useful tool for condensing a large set of variable data into main variables that capture the most important characteristics of the analyzed data [57].

For electron microscopy, 15 synapses of different visually identified types were analyzed using the SynapsEM method [58], which outputs numerical data on the number, diameter, and distribution of synaptic vesicles relative to the synaptic membrane and active zones. The size-related data were then imported into SPSS version 29.0.2.0 (IBM, Armonk, NY, USA) and statistically analyzed using the established boxplot method [59,60,61]. A graphical representation of the plots was used to present the data in Figure 8 d,f,h, and the data for all synapses are available upon request from the corresponding author. The observed distribution data were plotted for the position of the vesicles with respect to the plasmatic membrane and can be seen in Figure 8f,h,j. Synaptic gaps were measured using the interedge distance macro version 2.0 for Image J (version 2.14.0) [62]. The basic statistical analysis is provided directly by the macro.

## 5. Conclusions

This paper used a systematic approach of classical histological methods, immunofluorescence, and electron microscopy of human laryngeal tissue, enabling us to demonstrate the presence of a specialized neuromuscular system within the TA muscle (vocalis muscle) that is significantly more complex than previously thought. All structures, whether muscular or neural, show adaptations to the special function of the TA muscle (vocalis muscle). As such, the muscle fibers within the larynx have a myofibrillar structure that is capable of handling stress from multiple directions. The presence of an extensive mitochondrial system within the muscle fibers allows for sustained contractions. Muscle spindles are smaller and rare in the TA muscle (vocalis muscle). Synapses are widely distributed and of multiple ultrastructural types, suggesting both a motor and a sensory role for nerve fibers at this level. The synaptic features show the existence of a broader neuronal system within the TA muscle (vocalis muscle) than in other skeletal muscles. Combined with the specialized muscle fibers and the apparent presence of neurons within the TA muscle (vocalis muscle), this justifies the consideration that the muscle does not fit into the current classifications for muscle tissue and represents an intermediate, specialized muscle type. Future research should focus on expanding our understanding of the connection between the described structures and integrating the described results into the larger models of vocal fold vibration.

## Figures and Tables

**Table 1:** Antibodies and control tissues used.

Antibody	Manufacturer	Control Used	Location
Mouse anti-NF 1:50 MUB 1303p	Biozol	Rat brain	Eching, Germany
Goat Anti-ChAT 1:50 AB 144p	Sigma-Aldrich	Rat hypothalamus	Darmstadt, Germany
Mouse Alexa 488 conjugated Synaptophysin 1:100	Proteintech	Rat brain	Planegg-Martinsried, Germany
Goat anti-mouse Alexa 555 1:700	Invitrogen	Larynx, no primary antibody	Paisley Renfrewshire, UK
Rabbit anti-goat Alexa 488 1:1000	Invitrogen	Larynx, no primary antibody	Paisley Renfrewshire, UK
DAPI 1:1000	Invitrogen	Larynx, no primary antibody	Paisley Renfrewshire, UK

All slides were then analyzed and partially photographed using a Keyence BZ-X800 fluorescence microscope (Keyence Corporation, Osaka, Japan) and the corresponding software.

## Data Availability

All data included in this article are available on request from the corresponding author (R.-V.T.).
